# T-ALL and thymocytes: a message of noncoding RNAs

**DOI:** 10.1186/s13045-017-0432-0

**Published:** 2017-03-07

**Authors:** Annelynn Wallaert, Kaat Durinck, Tom Taghon, Pieter Van Vlierberghe, Frank Speleman

**Affiliations:** 1 Center for Medical Genetics, Ghent University, Ghent, Belgium; 2 Cancer Research Institute Ghent, Ghent, Belgium; 3 Department of Clinical Chemistry, Microbiology and Immunology, Ghent University, Ghent, Belgium

**Keywords:** T-ALL, Thymocytes, LncRNA, MicroRNA

## Abstract

In the last decade, the role for noncoding RNAs in disease was clearly established, starting with microRNAs and later expanded towards long noncoding RNAs. This was also the case for T cell acute lymphoblastic leukemia, which is a malignant blood disorder arising from oncogenic events during normal T cell development in the thymus. By studying the transcriptomic profile of protein-coding genes, several oncogenic events leading to T cell acute lymphoblastic leukemia (T-ALL) could be identified. In recent years, it became apparent that several of these oncogenes function via microRNAs and long noncoding RNAs. In this review, we give a detailed overview of the studies that describe the noncoding RNAome in T-ALL oncogenesis and normal T cell development.

## Background

### The noncoding RNAome

For decades, it was thought that only 2% of the human genome was functional given its coding potential for proteins. The remaining 98% of the genome was considered “junk DNA”. More recently, many studies have indicated that a large portion (up to 75% or more) of the human genome is actively transcribed while not coding for proteins [1]. These so-called noncoding RNAs consist of several distinct families including microRNAs, small nuclear RNAs, PIWI-interacting RNAs, and long noncoding RNAs. Interestingly, the detection of noncoding RNAs led to a solution for the G-value paradox that states that there is no correlation between the amount of coding genes and the complexity of the organism [2], while we do observe a correlation between the complexity of the organism and the ratio of the amount of noncoding genes to the total genomic DNA. This finding, indirectly, suggests that the increasing amount of noncoding RNA genes in the genome of organisms can account for their complexity [3]. In the last decade, the role of several of these noncoding RNA families has been intensively studied and key functions in both normal development and disease were determined.

#### MicroRNAs

The Ambros team described the identification of the first microRNA (miRNA) *lin*-*4* in *C. elegans* in 1993 [4]. However, only from the beginning of this century, the process of miRNA biogenesis was studied in depth showing that miRNAs play an important role in development and disease. MicroRNAs are short noncoding RNAs of approximately 22 nucleotides that function as post-transcriptional repressors of their target genes. MiRNA biogenesis starts with transcription by RNA Pol II of a primary miRNA (pri-miRNA), an RNA molecule consisting of one to several hairpin structures [5, 6]. This pri-miRNA is subsequently cleaved to one hairpin by the enzyme Drosha leading to the formation of a precursor miRNA (pre-miRNA), which is then translocated to the cytoplasm by Exportin-5 and processed by the Dicer complex to a mature double stranded miRNA [6–12]. In order to execute its gene regulatory function, one strand is incorporated into the “RNA-induced silencing complex” (RISC) thus guiding RISC to its target messenger RNA (mRNA) through complementary base pairing between the 3′ untranslated region (3′UTR) of the target mRNA and the miRNA. In most cases, this interaction will eventually lead to mRNA degradation or inhibition of protein translation (for further details on miRNA biogenesis, we refer to a review by Ha and Kim [13]). Remarkably, it has also been shown that the miRNA interaction with mRNAs during cell cycle arrest can recruit translation activators instead of translation repressors [14].

Typically, 3′UTRs of protein-coding genes harbor multiple bona fide seed sequences for different miRNAs. While the overall effect of a given miRNA on mRNA expression or translation may be modest, the action of multiple miRNAs on a single 3′UTR may significantly alter the expression of the gene. At the same time, the nature of this regulatory process creates the possibility of time and context-specific gene regulation, which, amongst others, is critical in normal development and cellular functions. As such, it is not surprising that microRNAs are implicated in various diseases, including cancer [15–17]. Indeed, miRNAs can act as oncogenes by repressing the expression of tumor suppressors in the cell. The prototypical miRNA oncogene is the *miR*-*17*∼*92* cluster (*oncomiR*-*1*) encompassing six different miRNAs that are overexpressed in several cancer entities [18]. This polycistron is directly activated by the MYC transcription factor or repressed by p53 [19–21] and controls a plethora of target genes including *PTEN*, *BIM*, and *p21* (*CDKN1A*), thereby broadly impacting on the phenotype of cells [22, 23]. One of the first described tumor suppressor miRNAs is encoded by the *miR*-*15a*/*16*-*1* cluster, and this locus is affected by recurrent 13q14 deletions in more than half of chronic lymphocytic leukemia (CLL) cases with the *BCL2* oncogene as a primary target [24, 25]. Following these landmark discoveries, many additional miRNAs have been identified to act as oncomirs or tumor suppressor miRNAs (reviewed in [16, 26, 27]). Recent studies also linked another class of small noncoding RNAs, the PIWI-interacting RNAs (piRNAs) to cancer. PiRNAs were detected to be upregulated in several cancer types, which could be linked to a poor prognosis. The specific mechanism of action of piRNAs in cancer biology should however be further investigated to find out if and how they are driving cancer development [28, 29].

Given the role for miRNAs in several cancer types and promising preclinical studies, further initiatives towards implementing miRNA-based therapies using miRNA mimics or miRNA antisense inhibitors are taken (e.g., Mirna Therapeutics—www.mirnatherapeutics.com and miRagen Therapeutics—www.miragentherapeutics.com). Remaining challenges are the risk of a miRNA to act as both an oncogene or tumor suppressor depending on the cancer type, off-target effects, and the bioavailability of miRNA mimics/inhibitors (reviewed in [30]). Further, miRNAs or miRNA signatures can be used for prognostic evaluation of cancer entities, as it was for example detected that miRNA expression profiles of acute myeloid leukemia (AML) patients clustered the samples in different groups that could be linked to cytogenetic risk categories [31].

#### Long noncoding RNAs

While the existence of certain long noncoding RNAs (lncRNAs) such as *XIST* (implicated in X-chromosome inactivation) has been known for some time [32, 33], the full recognition for lncRNAs as functionally relevant RNA molecules has only emerged more recently. Generally speaking, lncRNAs, in contrast to miRNAs, represent a functionally very heterogeneous group of RNA molecules that are defined by their length of at least 200 nucleotides and lack of protein-coding potential. While many lncRNA genes share characteristics with protein-coding genes in relation to splicing and polyadenylation, many lncRNAs are expressed at low levels and show poor species conservation compared to protein-coding genes. These characteristics have caused skepticism on the actual functional relevance of lncRNAs. On the other hand, their complex secondary and tertiary structures hint towards functional active molecules [34, 35], a notion that is also further supported by their remarkable tissue or cell type-specific expression pattern. Indeed, for an increasing number of lncRNAs, the normal function and its putative implication in certain diseases have been reported but for the vast majority of lncRNAs such functionalities remain to be discovered.

The total number of annotated lncRNAs is enormous and may exceed 100,000 transcripts [36]. LncRNAs can be classified according to their location and orientation relative to protein-coding genes. LncRNAs overlapping a protein-coding gene are categorized as “sense” or “antisense” depending on their transcriptional orientation compared to the protein-coding gene. “Intronic” lncRNAs are transcribed from an intron of another transcript, whereas “intergenic” lncRNAs are located between two coding genes without any overlap. A fifth category is represented by those lncRNAs that are transcribed on the opposite strand of a protein-coding gene, with the transcription start sites located less than 1 kb from each other. These lncRNAs are categorized as “bidirectional.” (Reviewed in [37]).

At present, in-depth insights into the function of specific lncRNAs are rather limited. These studies however illustrate the broad possible cellular functions of lncRNAs, with putative functions in transcription, shaping genome architecture or epigenetic regulation. Modulation (activation or repression) of transcription by lncRNAs can be either through binding and regulation of chromatin-modifying complexes (ex. PRC2 recruitment [38, 39]) or transcription factors [40] or by inhibition of the general transcription machinery [41, 42]. Also, a specific class of lncRNAs transcribed at enhancers, the so-called eRNAs, has been described [43, 44]. Post-transcriptionally, lncRNAs have been detected to aid mRNA processing and direct splicing [45, 46] and also effects on translation or mRNA degradation have been encountered 21307942. Some lncRNAs also have several binding sites for a miRNA. These lncRNAs are called competitive endogenous RNAs (ceRNAs) as they titrate miRNAs away from their conventional target mRNA [47–49] (Review on lncRNA functions in [50]).

As indicated above, long noncoding RNAs play a role in normal cell development but also in several types of heritable diseases and cancer [51]. One of the most well-characterized and described lncRNAs in cancer is the “metastasis-associated lung adenocarcinoma transcript 1” (*MALAT1*), a rather atypical lncRNA with a high expression and species conservation. *MALAT1* is expressed in nuclear speckles and plays a role in nuclear organization, transcription, and alternative splicing. It has been shown that *MALAT1* is upregulated in several cancer types, enhancing cancer metastasis, and high *MALAT1* expression is correlated with poor prognosis [52]. Another example of an oncogenic lncRNA involved in several cancer types is “homeobox transcript antisense RNA” (*HOTAIR*). It has been shown that *HOTAIR* functions in the recruitment of the “polycomb repressive complex 2” (PRC2) to specific loci in the genome, which leads to H3K27 trimethylation and transcriptional silencing of these loci [53].

In the last years, it was shown that a key subset of lncRNAs is expressed from enhancer sites [54]. Enhancers are loci on the genome that are bound by specific factors that modulate the transcriptional activity of a nearby gene. These loci are demarcated in the genome by specific histone modifications (e.g., H3K27ac and H3K4me1) and binding of key transcription factors (e.g., p300 and the Mediator complex). The RNA molecules expressed from these enhancers have been coined enhancer RNAs (eRNAs) and are between 50 and 2000 nucleotides in length. It is hypothesized that several eRNAs are necessary for the activity of enhancers, by recruiting transcription factors to the enhancers and aiding in the chromosomal looping to bring the enhancer bound transcription factors to the gene promoter [44, 55]. However, some concerns about the functionality of these eRNA transcripts arose, as it appears that only the act of transcription, but not the sequence, has an influence on the function of the enhancer [56, 57].

### T cell acute lymphoblastic leukemia

T cell acute lymphoblastic leukemia (T-ALL) is a hematological malignancy caused by oncogenic transformation of developing thymocytes. Normal T cell development is a strictly regulated process that occurs in the thymus. Immature thymocytes enter the thymus from the bone marrow and migrate through several thymic niches that drive specific stages of T cell development [58–68]. During these stages, specific markers are present at the membrane of these immature thymocytes and genomic rearrangements attribute to the formation of a functional T cell receptor, leading to a broad range of different mature T cell types characterized by a specific T cell receptor. During these stages of T cell development, abnormal activation of oncogenes or inactivation of tumor suppressor genes can lead to a differentiation arrest and an uncontrolled expansion of immature thymocytes evolving to fully transformed T-ALL lymphoblasts [69].

Different genetic lesions have been identified as driving events, marking specific subgroups of T-ALL with distinct gene expression patterns [70–74]: the *TAL*-rearranged subgroup, the *TLX1* subgroup, the *TLX3* subgroup, and the *HOXA*-overexpressing subgroup. Recently, a fifth subgroup with a poor prognosis, the immature T-ALL subgroup, has been added with an early T cell progenitor phenotype, but no single specific oncogenic driver event. This subgroup is marked by the overexpression of multiple oncogenic factors as *MEF2C*, *LMO2*, *LYL1*, and/or *HHEX* in several patients. To establish a full-blown leukemia, several other oncogenic effects cooperate with these subtype-specific driver events. For example, constitutive activation of the NOTCH1-signaling pathway is present in over half of all T-ALL patients, regardless of the subtype, indicating that hyperactive NOTCH1 signaling plays a central role in T-ALL biology [75]. The NOTCH-signaling cascade is necessary in the early stages of T cell development [58], but sustained NOTCH activation leads to the malignant transformation of thymocytes. For more in-depth information on T-ALL, we refer to several good reviews [72, 76–78].

Noncoding RNAs have been extensively studied in leukemia and normal hematopoiesis [79–85]; here, we will focus on the role of miRNAs and lncRNAs in T-ALL and T cell development.

## Methodological approaches in miRNA and long noncoding RNA research

### MicroRNAs

#### Analytical platforms

MiRNA expression studies have initially used RT-qPCR or microarray platforms, which enable simultaneous detection of several hundreds of miRNAs. More recently, advances in next-generation sequencing technology made it also possible to determine the expression profiles of miRNAs by means of small RNA sequencing. A major advantage of small RNA sequencing is that also novel miRNAs and isomiRs (miRNAs with small variations compared to a reference miRNA sequence) get detected [86, 87]. The recently published miRQC study gives a detailed overview of the strengths and weaknesses of the different miRNA detection methods and platforms [88].

#### In silico target gene prediction

After the identification of miRNAs of interest, their potential target mRNAs are usually identified based upon the miRNA seed sequence, a seven-nucleotide sequence mostly situated at positions 2–7 from the 5′-end that can interact through complementary basepairing with the 3′UTR of the miRNA. For this, several online tools can be used, including miRDB (mirDB.org) [89], miRanda (microRNA.org) [90], TargetScan (targetscan.org) [91] and the recently developed miSTAR (mi-star.org) [92]. These tools also enable the identification of all miRNAs that potentially target an mRNA of interest. The disadvantage of these *in silico* prediction algorithms is that they focus on the interaction between the 5′ miRNA seed sequence and the 3′UTR of the miRNA, but it has been shown that these interactions can also take place in the 5′UTR or coding sequence of the mRNA, that in only 60% of the cases the seed interactions are perfectly complementary (others contain bulged or mismatched nucleotides) and that sometimes the 3′-end and not the 5′-end of the miRNA is used for base pairing [93]. Furthermore, these methods do not take into account the site accessibility as other RNA-binding proteins might block the miRNA binding site [94].

#### Wet lab validation of miRNA target genes

Target prediction can also be achieved through several in vitro methods. High-troughput sequencing methods used for miRNA-mRNA interaction detection are for example HITS-CLIP (high-throughput sequencing of RNA isolated by crosslinking immunoprecipitation) [95] or PAR-CLIP (photoactivatable ribonucleoside-enhanced crosslinking and immunoprecipitation) [96], which are methods developed to identify the specific binding sites of RNA-binding proteins. In miRNA research, these are specifically used to pull down the RNA that interacts with proteins from the RISC-complex, mostly AGO2. By comparing the pulled down RNA after overexpression or knockdown of a miRNA with a mock control, the exact interaction partners of a miRNA can be identified [96–98]. Next to these methods, all miRNA-mRNA interactions can be directly mapped using the CLASH (crosslinking, ligation, and sequencing of hybrids) technology [93]. In this method, AGO-associated miRNA-target duplexes are ligated, resulting in a chimeric RNA molecule that is subsequently sequenced immediately revealing the exact miRNA-mRNA interaction site [93]. While these methods obtain valuable novel information on miRNA targets, they are however labor intensive and technically challenging. An overview of other in vitro methods can be found in the review by Thomson et al. [94].

To validate potential miRNA-mRNA interactions, a luciferase reporter assay is the method of choice. In this assay, the 3′UTR of the target mRNA is cloned next to a reporter gene (e.g. luciferase) and a functional miRNA-mRNA interaction should result in a decrease of the reporter gene signal after overexpression of the miRNA of interest. A direct interaction between the miRNA and the 3′UTR of the target gene could then be confirmed if the decrease in signal is rescued by mutations of the miRNA binding sites.

This reporter assay has also been applied in 3′UTR library screens in order to detect possible interactions of known miRNAs with a certain gene of interest. In such 3′UTR library screen, a plasmid containing the luciferase report gene with the 3′UTR of the gene of interest and a miRNA library are transfected together in HEK293T cells [99]. Subsequent screening of the luciferase signal intensity allows for the identification of potential functional miRNA-mRNA interactions, which also need to be validated through subsequent mutagenesis assays.

#### In vivo studies of miRNA function in T-ALL development

*NOTCH1*-activating mutations are frequently detected in human T-ALL and it has been shown that *NOTCH1* serves as a potent oncogene that can drive T-ALL development in mice. To study the role of miRNAs in T-ALL in vivo, a NOTCH1-sensitized mouse model was used [100]. To establish this model, fetal liver cells with hematopoietic progenitor cells (HPCs) are isolated from pregnant mice. These HPCs are then transduced with *ICN1* (active NOTCH1) and a vector containing an antagomiR or premiR. After irradiation of the recipient mice, these transduced HPCs are injected by tail vein injection. The leukemia onset of these mice, compared with control mice (*ICN1* + a negative control miRNA), gives an indication of the oncogenic or tumor suppressive potential of the tested miRNA.

Junker et al. showed that the leukemia onset of this mouse model is dependent on *Dicer1*-mediated biogenesis of miRNAs [101]. When HPCs with ICN1 overexpression were injected in conditional *Dicer1* knockout mice, where *Dicer1* is inactivated when thymocytes or leukemic cells transit from the double negative to double positive (CD4^+^CD8^+^) stage, there was no leukemic onset compared with control mice that all developed leukemia in less than 100 days.

### LncRNAs

#### Analytical platforms

In some early studies, lncRNAs have been investigated using dedicated microarrays [102, 103]. More recently, RNA sequencing has become the method of choice, particularly given the significant tissue and spatial specific expression of lncRNAs. RNA-sequencing detection of lncRNAs requires a higher read depth in comparison to protein-coding mRNAs, given the low expression levels of most lncRNAs [104]. In addition to standard poly(A) RNA sequencing, total RNA sequencing (with ribosomal RNA depletion) is the preferred sequencing technique for more exploratory studies, as it appears that several lncRNAs do not have a poly(A) tail [105]. The major advantage of RNA sequencing is the ability to detect novel lncRNAs or different splicing variants of a known lncRNA [104, 106].

#### Guilt-by-association analysis

One of the major challenges in lncRNA research is the selection of candidate lncRNAs for further functional studies. Guilt-by-association analysis has been applied to detect potential pathways in which a certain lncRNA of interest is involved [107, 108]. This analysis is based on the correlation of the candidate lncRNA expression pattern in a sufficient large number of (patient) samples to the expression of all protein-coding genes. Strong positive and/or negative correlations between the lncRNA and several protein-coding genes could hint towards the involvement of the lncRNA in the same pathways as these protein-coding genes.

#### In vitro studies of lncRNAs

The cellular localization of the lncRNA can be determined by means of RNA-FISH (fluorescence in situ hybridization) or cell fractionation. Nuclear lncRNAs are probably involved in gene regulation or splicing control, whereas cytoplasmic lncRNAs might have a plethora of other functions such as miRNA sequestration, regulation of translation, or protein complex formation. To detect the interaction of the lncRNA with DNA, other RNAs, or proteins, several techniques have been published. These are based on the use of biotinylated oligonucleotides complementary to the RNA of interest to pull down its associated DNA, RNA, or proteins (ChIRP, chromatin isolation by RNA purification [109]; CHART, capture hybridization analysis of RNA targets [110]; RAP, RNA antisense purification [111]). On the other hand, lncRNAs that are interacting with a protein of interest can be detected by means of RIP (RNA immunoprecipitation) [112]. These technologies and many more are nicely reviewed by Chu et al. [113]. Furthermore, the change in transcriptional profiles after lncRNA knockdown could already hint towards potential roles for the lncRNAs. However, it should be noted that knockdown of lncRNAs is not always as straightforward as for protein-coding genes. One major disadvantage is the nuclear location of several lncRNAs, which makes knockdown by siRNAs less efficient. The use of antisense oligonucleotides (ASOs) could be a solution for this problem as ASOs activate the RNaseH mechanism in the nucleus to cut the RNA target. The use of the cluster of regularly interspaced short palindromic repeats (CRISPR)/Cas9 technology to knockout lncRNAs also imposes some obstacles, as lncRNAs might overlap with protein-coding genes (sense or antisense) or with regulatory elements (ex. enhancers). CRISPRi [91, 114], using an inactivated Cas9 protein linked to a transcription repressor, could be a possible solution to inhibit the expression of the lncRNA. Here, the guide RNA is targeted to the transcription start site of the lncRNA, inhibiting its expression.

#### In vivo studies of lncRNAs

The lack of sequence conservation of lncRNAs between human and mice makes it very difficult to find orthologous lncRNAs for in vivo studies. However, for several lncRNAs, the preservation of secondary structures, sequence domains, or interacting proteins could be detected, as reviewed by Johnsson et al. [35]. One remarkable feature detected by several groups is that the promoter of lncRNAs showed a higher degree of sequence conservation than the exons [104, 107, 115, 116]. This topic is also reviewed by I. Ulitsky [117]. One way to study the oncogenic potential of lncRNAs in vivo without the knowledge of the mouse orthologous lncRNA is the use of xenografts by implanting human cell lines in mice. These cell lines could be modulated by means of knockdown or overexpression of the lncRNA and cancer progression could be monitored. In T-ALL, a competition assay could be used where wild type and modulated cell lines with specific fluorescent markers are mixed and consequently injected in mice. After a few weeks, the fluorescent signal ratios can then be measured by flow cytometric analysis of the blast cells [118].

## Oncomirs and tumor suppressor miRNAs in T-ALL

### T-ALL miRNA oncogenes

*MiR*-*19b* was one of the first oncogenic miRNAs described in T-ALL by the Wendel team [119]. This miRNA is part of the abovementioned *mir*-*17*~*92* cluster. The oncogenic role of the cluster in T-ALL was strongly suggested through the finding of a new translocation t(13;14)(q32;q11) that juxtaposed the *miR*-*17*~*92* cluster to the *TCRA*/*D* locus thereby placing it under the immediate control of the strong *TCRA/D* enhancer. This translocation occurred together with a t(9;14)(q34;q11) translocation that contributes to the aberrant activation of the *NOTCH1* gene. The coexistence of these two translocations hinted towards the collaboration of *NOTCH1* and the *miR*-*17*~*92* cluster in T-ALL development. In order to define which members of the cluster effectively contributed to T-ALL formation, cytokine-dependent FL5-12 lymphocytes were transduced with individual miRNAs of the cluster followed by IL-3 withdrawal. In these assays, *miR*-*19b* showed the strongest oncogenic capacity, which is in line with the fact that *miR*-*19b* shows the highest expression of all members of the *miR*-*17*~*92* cluster in human T-ALL. A *NOTCH1*-sensitized mouse model was subsequently used to confirm the oncogenic role of *miR*-*19b* in vivo. Finally, target prediction algorithms in combination with functional validation experiments identified different components of the PI(3)K signaling pathway as direct *miR*-*19b* targets, including *PP2A*, *PRKAA1*, *BIM*, and *PTEN*.

A few years later, Ye et al. performed a large bio-informatics screening to point out central hubs in the T-ALL network [120]. To this end, combinations of genes and miRNAs known to be involved in T-ALL were tested with target prediction algorithms. Furthermore, possible transcription factor regulatory relationships (feed forward and feedback loops) were determined based on predicted transcription factor binding sites near T-ALL genes and miRNAs. This led to a complex network that contained 21 T-ALL genes, 21 T-ALL miRNAs, and 28 transcription factors. The main hubs in this *in silico* established network contained 4 miRNAs of the *miR*-*17*~*92* cluster, again revealing an important role for this cluster in T-ALL. In addition, these authors revealed that *miR*-*19* could regulate NF-κB signaling through direct targeting of *CYLD*.

In 2011, a more in-depth study was performed towards identifying oncogenic miRNAs targeting known tumor suppressor genes in T-ALL [121]. In this study, miRNA expression data was compared with an unbiased miRNA library screen, computational target prediction analyses and in vivo modeling to identify the most promising candidates. Eventually, this resulted in the identification of a network of five oncogenic miRNAs (*miR*-*19b*, *miR*-*20a*, *miR*-*26a*, *miR*-*92*, and *miR*-*223*), which shared a panel of direct tumor suppressor target genes previously implicated in T-ALL biology (*IKZF1*, *PTEN*, *BIM*, *PHF6*, *NF1*, and *FBXW7*). MiRNAs with the same target genes also showed a cooperative effect on cell viability. Three of these miRNAs (*miR*-*19b*, *miR*-*20a*, and *miR*-*92*) belong to the oncogenic *miR*-*17*~*92* cluster, whereas *miR*-*223* was subsequently shown to be activated by TAL1 [122, 123] and NOTCH1 [124], two important T-ALL oncogenes (discussed below), further supporting the original observations of this study.

In subsequent studies, additional miRNAs with an oncogenic role in the development of T-ALL have been reported. *MiR*-*128*-*3p* is highly expressed in T-ALL patients and has increased expression in T-ALL samples compared to healthy donor thymocytes. *MiR*-*128*-*3p* directly inhibits the expression of the tumor suppressor *PHF6* and overexpression caused accelerated leukemia onset in the *NOTCH1*-sensitized mouse model [99]. *MiR*-*21* is highly expressed in both murine and human T-ALL and is involved in the inhibition of apoptosis, probably by regulating *Pdcd4*, known to play a role in the apoptosis pathway by inhibition of *BCL*-*xL* translation [101]. Another example is *miR*-*142*-*3p*, which is upregulated in T-ALL patient samples compared to thymocytes of healthy donors and is one of the top expressed miRNAs in T-ALL [121, 125]. *MiR*-*142*-*3p* plays a role in cell proliferation through an indirect inhibition of *cAMP* (cyclic AMP) and *PKA* (protein kinase A), an inhibitor of T cell leukemia proliferation. Furthermore, *miR*-*142*-*3p* directly targets glucocorticoid receptor alpha (*GRα*), with high *miR*-*142*-*3p* levels being involved in glucocorticoid resistance, and is linked to poor prognosis [125]. *MiR*-149* was detected as being upregulated in T-ALL cell lines and bone marrow of T-ALL patients in comparison to peripheral blood. This miRNA promotes cell proliferation and reduces cell apoptosis and might perform this oncogenic function by its direct targeting of *JunB* [126].

Finally, other studies made use of correlation analyses between miRNA and mRNA expression in T-ALL patient samples to detect potential novel oncomiRs. For example, the expression of *miR*-*590* is negatively correlated with *RB1* expression and it was found that *miR*-*590* plays an oncogenic role in cell proliferation and migration and invasion, by directly targeting *RB1* [127]. A second example is the negative correlation between *miR*-*181a* and *EGR1*, a tumor suppressor in several other cancer entities. The *miR*-*181a*/*EGR1* pair probably has a role in cell cycle regulation [128]. *miR*-*181a* is also linked to the NOTCH1 signaling pathway which is discussed further in more detail [129].

### T-ALL tumor suppressor miRNAs

The Wendel team also conducted a screening for miRNAs with a tumor suppressor function [130]. They selected abundantly expressed miRNAs in thymocytes from healthy donors that had at least a 10-fold lower expression in primary T-ALL samples. Further selection was performed by in vitro proliferation assays after overexpression of the miRNAs. This approach eventually led to the identification of five miRNAs (*miR*-*29*, *miR*-*31*, *miR*-*150*, *miR*-*155*, and *miR*-*200*) with tumor suppressive effects in vitro and in vivo. To identify the potential mRNA targets by which these miRNAs performed their tumor suppressive effect, predicted targets with higher expression in T-ALL patients as compared to healthy donors were selected, in keeping with a potential oncogenic function of the targets. The known T-ALL oncogene *MYB* (for *miR*-*150*, *miR*-*155*, and *miR*-*200*) and also a potential new oncogene in T-ALL, *HBP1* (for *miR*-*29*, *miR*-*31*, *miR*-*155*, and *miR*-*200*) appeared to be key targets of this tumor suppressive miRNA network. Remarkably, it was also shown that the oncogenic NOTCH1/c-MYC pathway inhibited the expression of *miR*-*31*, *miR*-*150*, and *miR*-*155*.

To further evaluate the post-transcriptional regulation of the T-ALL oncogene *MYB* by miRNAs, the Speleman team performed a miRNA library screen testing the putative interaction of 470 miRNAs with the 3′UTR of *MYB* by a luciferase reporter assay. Combined with mRNA and miRNA expression profiling data from 64 T-ALL patient samples, *miR*-*193b*-*3p* was detected as a direct negative regulator of *MYB. MiR*-*193b*-*3p* was also lower expressed in TAL-rearranged T-ALL patients, in keeping with *MYB* upregulation in this T-ALL genetic subtype. Importantly, inhibition of *miR*-*193b*-*3p* in the *NOTCH1*-sensitized mouse model significantly increased leukemia onset [131].

In another study, *miR*-*204* was detected as a potential tumor suppressive miRNA as it was lower expressed in T-ALL patient samples compared to normal T cells from peripheral blood. This was further supported by the observation that *miR*-*204* could inhibit proliferation, migration, and invasion of T-ALL cell lines and directly targets *SOX4*, a protein involved in tumorigenesis of AML [132].

### T-ALL subtype-specific miRNAs

As indicated above, T-ALL samples can be classified in different genetic subtypes, which display unique gene expression signatures [70–74]. Although different studies have linked miRNAs to specific genetic subtypes of human T-ALL, a comprehensive study on the expression of subtype-specific miRNAs in human T-ALL remains to be accomplished.

One of the first papers that described miRNAs in T-ALL linked high expression of the *miR*-*17*~*92* cluster to *TLX1*, *TLX3*, and *NKX2*-*5* overexpressing T-ALL primary samples and cell lines. This miRNA cluster seems to be activated by these transcription factors and imposes increased cell survival through the inhibition of *E2F1* [133].

Schotte et al. linked *miR*-*196b* to the HOXA-overexpressing subtype with MLL-rearrangements, *CALM*-*AF10*, or *SET*-*NUP214* fusions or inversion on chromosome 7 [134]. Since *miR*-*196b* is located in the *HOXA*-locus, this link might be due to co-activation. High expression of *miR*-*196a* and *miR*-*196b* was subsequently also linked to T-ALL samples with an early immunophenotype and concomitant expression of CD34 and CD33 [135].

Furthermore, *miR*-*223* has been linked to a myeloid-like T-ALL phenotype [136] but has also been identified as a target of the TAL1 transcription factor oncogene [122, 123]. Moreover, high expression of *miR*-*221* and *miR*-*222* has been linked to the poor prognostic subtype of human early T cell precursor acute lymphoblastic leukemia (ETP-ALL), and it was discovered that miR-222 directly inhibits the expression of the proto-oncogene *ETS1* [137]. In the same study, *miR*-*19a* and *miR*-*363* were detected as specifically downregulated in ETP-ALL.

### MiRNAs in the NOTCH1 regulatory network

As mentioned in the introduction, *NOTCH1*-activating mutations are present in over half of all T-ALL patients. A plethora of canonical NOTCH1 downstream protein-coding targets have been described over the last decade. More recently, it became apparent that also miRNAs play a role in the NOTCH1 regulatory network in the context of T-ALL development.

Li et al. described *miR*-*451* and *miR*-*709* as possible tumor suppressor miRNAs in murine T-ALL. These miRNAs are downregulated in T-ALL and show a dynamic expression pattern during normal T cell development. The tumor suppressor role of these miRNAs was further established by a delayed leukemia onset after overexpression of *miR*-*451* or *miR*-*709* in the *NOTCH1*-sensitized mouse model. *MiR*-*451* and *miR*-*709* directly target *c*-*Myc*, a known oncogene activated by NOTCH1 in T-ALL. Next to *c*-*MYC*, *miR*-*709* also directly targets *Ras*-*GRF1* and *Akt*. Motif analysis followed by ChIP-sequencing revealed the positive regulation of these miRNAs by *E2A*, which itself is inhibited by NOTCH1 signaling. The NOTCH1/miR-451/c-MYC axis also plays a role in human T-ALL (*miR*-*709* has no human homologue) [138].

Later, this network was further expanded by adding a feed forward loop between *NOTCH1* and *c*-*MYC* that was regulated by the tumor suppressive miRNA, *miR*-*30a* [139]. The expression of *miR*-*30a* is lower in T-ALL patient samples with hyperactive NOTCH1 compared to *NOTCH1* wild-type cases. NOTCH1 signaling activates the expression of *c*-*MYC* and *c*-*MYC* inhibits *miR*-*30a* expression [140]. Target prediction analysis and reporter assays then demonstrated that *miR*-*30a* targets *NOTCH1*. This implies that oncogenic activation of *NOTCH1* leads to an overexpression of *c*-*MYC*, followed by a *miR*-*30a* downregulation. This then releases the inhibition of *NOTCH1* expression by *miR*-*30a* [139].

It has also been shown that NOTCH1-induced murine T-ALL development was hampered by the deletion of the *miR*-*181a*-*1*/*b*-*1* gene. Remarkably, the effects of *miR*-*181a*-*1*/*b*-*1* change depending on the expression level of *Notch1*. If the expression of *Notch1* is high, the deletion of the *miR*-*181a*-*1*/*b*-*1* gene strongly delays T-ALL development, whereas the deletion leads to a full inhibition of T-ALL if *Notch1* expression is lower. *miR*-*181a* regulates Notch signaling by inhibition of *Nrarp*, which is a negative regulator of the NOTCH1 downstream signaling. Furthermore, *miR*-*181a* was also necessary in early T cell development, where it inhibits negative regulators of pre-T cell receptor signaling (ex. *Dusp5* and *Dusp6*) [129].

*MiR*-*223* was detected as differentially expressed in murine Notch-modulated T-ALL models. Motif analysis and ChIP-sequencing showed the binding of the ICN1 complex and NF-κB to the promoter of *miR*-*223*, which leads to the activation of transcription of this miRNA. *MiR*-*223* itself further negatively regulates *FBXW7*, a known tumor suppressor gene in T-ALL. In contrast to this finding, γ-secretase inhibitor (GSI) treatment (which inhibits downstream NOTCH1 signaling) showed upregulation of *miR-223* in GSI-resistant T-ALL cell lines [124]. These contradictory results could later be explained by the activation of C/EBPα after GSI treatment, which can activate *miR*-*223* as well [141]. *MiR*-*223* is also important in the TAL1 downstream pathway, which will be discussed in the next paragraph.

### MiRNAs up- and downstream of the TAL1 oncogene

*TAL1*/*SCL* overexpression is one of the major oncogenic events in T-ALL, which could delineate a specific T-ALL subtype. Mansour et al. studied the downstream miRNAs of TAL1 [122]. In this study, *miR*-*223* was the most promising candidate as it was most strongly differentially expressed upon *TAL1*-knockdown and direct binding of *TAL1* to the *miR*-*223* promoter was shown. Next to that, *TAL1*-positive T-ALL cells needed *miR*-*223* for their sustained cell survival. They also showed that the expression of *TAL1* and *miR*-*223* is strongly correlated during normal T cell development, implicating that the expression of *miR*-*223* is high in early T cell progenitors and low from the DN3a stage onto more mature T cell stages. Furthermore, it was proven that *miR*-*223* directly inhibits the expression of *FBXW7* and in this way supports the oncogenic function of *TAL1* [121, 122]. By means of TAL1-overexpression, Correia et al. also showed the direct activation of *miR*-*223* but also direct repression of *miR*-*146b*-*5p* by TAL1. Direct or indirect TAL1-regulated miRNAs were predicted (by *in silico* analysis) to target several genes in the *TAL1* downstream pathways [123].

Because several T-ALL patients show *TAL1* overrexpression without a known cause, Correia and colleagues hypothesized that the downregulation of miRNAs that target *TAL1* might be a novel oncogenic event in T-ALL. Target prediction algorithms revealed several miRNAs with potential binding sites in the 3′UTR of *TAL1*. Five of these miRNAs (*miR*-*101*, *miR*-*520d*-*5p*, *miR*-*140*-*5p*, *miR*-*448*, and *miR*-*485*-*5p*) could be validated as direct inhibitors of *TAL1*, of which four miRNAs (not miR-520d-5p) where lower expressed in T-ALL patient samples compared to normal bone marrow cells [142].

**Fig. 1:**
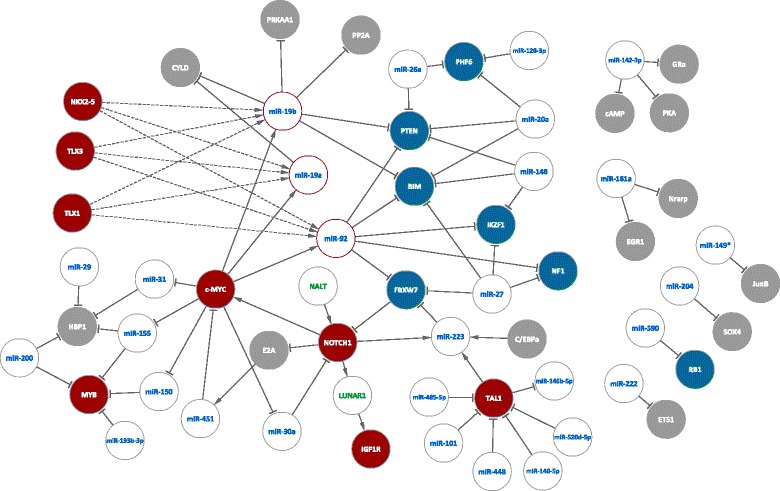
Overview of the noncoding RNAs implicated in T-ALL. miRNAs (*blue text*) and lncRNAs (*green text*) studied in T-ALL oncogenesis. The mRNAs linked to the ncRNAs are annotated in the filled circles. Bona fide oncogenes and tumor suppressor genes in T-ALL are annotated in respectively *red* and *blue background. Dashed lines* represent indirect interactions. miRNAs of the miR-17~92 cluster are highlighted with a *red circle*

An overview of the described miRNA-mRNA interaction can be found in Fig. 1 and in Table 1.

**Table 1:** miRNAs implicated in T-ALL biology

	miRBase release 21	Function	Direct targets	Refs
miR-19a	hsa-miR-19a-3p	ONC	CYLD	[120, 137]
		ETP low		
miR-19b	hsa-miR-19b-3p	ONC	BIM, CYLD, PP2A, PRKAA1, PTEN	[119–121]
miR-20a	hsa-miR-20a-5p	ONC	BIM, PHF6, PTEN	[121]
miR-21	hsa-miR-21-5p	ONC	PDCD4	[101]
miR-26a	hsa-miR-26a-5p	ONC	BIM, PHF6, PTEN	[121]
miR-29	hsa-miR-29a-3p	TSG	HBP1	[129]
miR-30a	hsa-miR-30a-5p/3p	MYC repressed	NOTCH1	[139, 140]
		Targets NOTCH1		
miR-31	hsa-miR-31-5p	TSG	HBP1	[130]
miR-92	hsa-miR-92a-3p	ONC	BIM, FBXW7, IKZF1, NF1, PTEN	[121]
miR-101	hsa-miR-101-3p	Targets TAL1	TAL1	[142]
miR-128-3p	hsa-miR-128-3p	ONC	PHF6	[99]
miR-140-5p	hsa-miR-140-5p	Targets TAL1	TAL1	[142]
miR-142-3p	hsa-miR-142-3p	ONC	cAMP, GRα, PKA	[125]
miR-146b-5p	hsa-miR-146b-5p	TAL1 repressed	–	[123]
miR-149*	hsa-miR-149-3p	ONC	JunB	[126]
miR-150	hsa-miR-150-5p	TSG	MYB	[130]
miR-155	hsa-miR-155-5p	TSG	HBP1, MYB	[130]
miR-181a	hsa-miR-181a-5p	ONC	EGR1, NRARP	[128]
miR-193b-3p	hsa-miR-193b-3p	TSG	MYB	[131]
		TAL-R low		
miR-196a	hsa-miR-196a-5p	IMM high	ERG	[135]
miR-196b	hsa-miR-196b-5p	HOXA high	ERG	[134, 135]
		IMM high		
miR-200c	hsa-miR-200c-3p	TSG	HBP1, MYB	[130]
miR-204	hsa-miR-204-5p	TSG	SOX4	[132]
miR-221	hsa-miR-221-3p	ETP high	–	[137]
miR-222	hsa-miR-222-3p	ETP high	ETS1	[137]
miR-223	hsa-miR-223-3p	ONC	FBXW7	[121–124, 136]
		Myeloid high		
		TAL-R high		
		NOTCH1 activated		
		TAL1 activated		
miR-363	hsa-miR-363-3p	ETP low	–	[137]
miR-448	hsa-miR-448	Targets TAL1	TAL1	[142]
miR-451	hsa-miR-451a	NOTCH1 repressed	c-MYC	[138]
miR-485-5p	hsa-miR-485-5p	Targets TAL1	TAL1	[142]
miR-520d-5p	hsa-miR-520d-5p	Targets TAL1	TAL1	[142]
miR-590	hsa-miR-590-5p	ONC	RB1	[127]
miR-92	hsa-miR-92a-3p	ONC	BIM, FBXW7, IKZF1, NF1, PTEN	[121]

The most recent miRBase annotation was retrieved using the miRBase Tracker, www.mirbasetracker.org [160]

*ONC* oncogenic miRNA, *TSG* tumor suppressor miRNA, *ETP* ETP-ALL, *TAL-R* TAL-rearranged T-ALL, *IMM* immature T-ALL, *HOXA* HOXA-overexpressing T-ALL

## Long noncoding RNAS implicated in T-ALL

In contrast to miRNAs, lncRNAs emerged more recently on the cancer scene and fewer publications have been published so far. The possible functions of lncRNAs are most probably very diverse as exemplified by those described so far in the lncRNA field. Moreover, modulating lncRNAs and identifying their function can be notoriously difficult and require extensive investigations.

### NOTCH1-driven lncRNAs

The first comprehensive study of lncRNAs in T-ALL comprised mRNA and lncRNA expression profiles of T-ALL cell lines and primary T-ALL patient samples by means of deep total RNA sequencing. Direct NOTCH1-regulated lncRNAs were determined by pharmacological inhibition of the NOTCH1 pathway by means of GSIs in two T-ALL cell lines and by NOTCH/RBPJκ ChIP-sequencing. Trimarchi et al. prioritized *LUNAR1* (leukemia-induced noncoding activator RNA 1) as a NOTCH1-induced candidate oncogenic lncRNA for further functional analysis. This was based on its strong correlated expression with *IGF1R*, as *IGF1R* was already previously linked to T-ALL development. In addition, the *LUNAR1* locus is characterized by an active promoter based on the chromatin structure as determined in cell lines with hyperactive NOTCH1 signaling and the transcript structure of *LUNAR1*, as determined by “rapid amplification of cDNA ends” (RACE), has no protein-coding potential. Hi-C and 3C (chromosome conformation capture) proved a physical interaction between the *LUNAR1* promoter and an active enhancer in the last intron of its neighboring gene *IGF1R*. Also, knockdown of *LUNAR1* led to a decrease in expression of *IGF1R*, whereas overexpression of *LUNAR1* did not have any effect on *IGF1R*, in keeping with a *cis*-acting role of *LUNAR1*. Next, in-depth in vitro and in vivo experiments could unravel the mechanism by which *LUNAR1* has an oncogenic role in T-ALL. Xenograft assays with a mix of human T-ALL cells with or without knockdown of *LUNAR1* revealed tumors with a significant loss of representation of cells where *LUNAR1* was depleted, again proving an oncogenic role of *LUNAR1* in T-ALL development. On a molecular level, Trimarchi and colleagues could show that *LUNAR1* is involved in the recruitment of the Mediator complex and RNA Pol II to the enhancer located in the last intron of *IGF1R*, leading to full transcriptional activation of the *IGF1R* gene [118].

In a parallel study, the repertoire of NOTCH1-driven lncRNAs in T-ALL was further unraveled by Durinck et al., through characterization of lncRNAs of which the expression was affected by GSI treatment of T-ALL cell lines and under control of NOTCH signaling in CD34^+^ thymocytes [61, 102]. By means of RNA - sequencing, a set of known and novel lncRNAs that are directly regulated by NOTCH1 in both normal and malignant T cell development was identified, with one of the most prominent NOTCH1 candidate lncRNAs apparent from both in vitro model systems being the previously described *LUNAR1*. Integration of the obtained RNA-seq profiles of GSI-treated cell lines and NOTCH1-stimulated CD34+ T cell progenitors with NOTCH1 ChIP-sequencing profiles showed that the majority of the identified NOTCH1-regulated lncRNAs showed ICN1 binding in the vicinity of their promoter. In addition, a subset of those was also bound by MED1 and BRD3, hinting towards a potential role of enhancer RNAs for a subset of the identified NOTCH1-regulated lncRNAs [102].

In addition to the above studies focusing on NOTCH1-controlled lncRNAs, yet another investigation identified *NALT* (Notch1-associated lncRNA in T-ALL) as a lncRNA involved in the regulation of *NOTCH1* expression. It is located 400 bp upstream of the *NOTCH1* locus in the antisense direction and is higher expressed in T-ALL patient bone marrow compared to healthy control samples. In vitro and in vivo knockdown experiments could further show a potential role for *NALT* as a transcriptional activator involved in cell proliferation [143].

### T-ALL subtype-specific lncRNAs

As indicated above, gene expression studies have been shown to allow genetic subgroup classification. To explore this for lncRNA expression profiles, the Speleman team screened a cohort of 64 primary T-ALL patient samples for expression of all protein-coding genes and 13,000 lncRNAs [144]. This cohort consisted of 15 immature, 17 *TLX1*/*3*, 25 *TAL*-rearranged, and 7 *HOXA*-overexpressing T-ALL cases. This study allowed defining subsets of lncRNAs specific for each of the T-ALL genetic subtypes. Furthermore, the authors linked the lncRNA expression pattern in these T-ALL subtypes to the different stages of healthy T cell development in the thymus. As the immature T-ALL subtype lymphoblasts occur from a differentiation arrest early during T cell development (CD34^+^ thymocytes), it appeared that several lncRNAs that are upregulated in the immature T-ALL subtype are also higher expressed in the CD34^+^ thymocytes compared to later stages during T cell development. These lncRNAs might be involved in normal T cell development. On the other hand, lncRNAs were identified in the immature T-ALL subtype group with significantly higher expression in immature T-ALL as compared to CD34^+^ thymocytes, revealing a potential oncogenic role during T-ALL development. The same comparisons could be made for the *TAL*-rearranged patients that resemble a later differentiation arrest during T cell development, the double positive CD4^+^CD8^+^ stage [103].

## Noncoding RNAs in T cell development

**Fig. 2:**
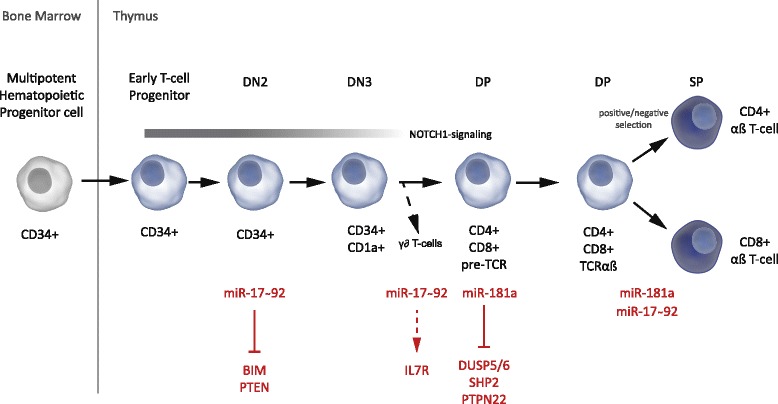
MicroRNAs involved in T cell development in the thymus. Immature T cells migrate from the bone marrow to the thymus where they go through several stages of differentiation (early T cell prognitors (ETP), double negative T cells (DN), double positive T cells (DP) and single positive T cells (SP)), which are marked by different membrane receptors (CD34, CD1a, CD4, CD8, TCR, etc). Mature T cells leave the thymus as either CD4+ or CD8+ αβ T cells or γδ T cells and perform several functions in the immune defense of the body. MicroRNAs play a role during this process, with proven function for the miR-17~92 cluster and miR-181a

Normal thymopoiesis is a tightly regulated developmental process that is initiated with CD34^+^ early T cell progenitors that migrate from the bone marrow towards the thymus. Within this thymic microenvironment, discrete developmental stages of T cell development can be identified through a combination of cell surface markers (CD34, CD4, CD8, CD3, etc.) and each of these stages contains a distinct transcriptional profile [58–68] (Fig. 2). As noncoding RNAs show a very tissue and cell type-specific expression pattern, the possible involvement of miRNAs and lncRNAs in the clearly distinct steps of T cell development is quite obvious.

### MicroRNAs in T cell development

Several miRNAs have been discovered over the last years that are involved in normal T cell development in the thymus. Their overall relevance was nicely illustrated by a study by Cobb et al. that showed that deletion of *Dicer* in early T cell progenitors in mice led to a decrease in thymic cellularity as a consequence of reduced survival of the αβ T cell lineage, thus revealing a role for miRNAs in the double negative to double positive stage transition [145, 146]. In addition, miRNA processing by Dicer is also necessary for the positive selection of thymocytes and the transition from the double positive to the CD8^+^-single positive stage, as shown in conditional *Dicer* knockout mice with a CD4-Cre transgene [147]. It has also been shown that miRNAs and even isomiRs change in expression during T cell development, indicating that not only the expression but also the processing of the miRNAs is altered during thymopoiesis [148].

Despite their clear importance, data on the role of individual miRNAs is rare. *MiR*-*181a*, however, not only plays a role in *NOTCH1*-driven T-ALL but also appears to be involved during normal T cell development. The expression of *miR*-*181a* is high at the double positive T cell stage and decreases during development, with almost no expression in differentiated T cells [149, 150]. Furthermore, it has been shown that *miR*-*181a* increases thymocyte sensitivity by directly inhibiting the expression of *DUSP5*, *DUSP6*, *SHP2*, and *PTPN22* which are negative regulators of TCR signaling [151]. *miR*-*181a* also appears to be involved in the regulation of positive and negative selection of thymocytes [150, 151] (Fig. 2).

The *miR*-*17*~*92* cluster inhibits the expression of *PTEN* and of the pro-apoptotic protein BIM, which leads to T cell survival at the DN2 stage of T cell development [152]. It has also been shown that the *miR*-*17*~*92* cluster is necessary for cell survival at the double negative to double positive transition of T cell development by regulating the IL7R receptor surface expression and the response to IL-7 [153]. Furthermore, this cluster is also involved in positive and negative selection of thymocytes [152]. Recently, it has been shown that the expression of the *miR*-*17*~*92* cluster is regulated by TCR signaling and, in this way, indirectly by *miR*-*181a*. The expression of *miR*-*17*~*92* can inhibit CD69 expression, which is also activated by TCR signaling. With this feed forward loop, cell-to-cell variation in the thymocytes is regulated. This further marks the importance of miRNAs during normal T cell development [154] (Fig. 2).

### LncRNAs in T cell development

As lncRNAs are known to be expressed tissue specifically, it should be no surprise that also lncRNAs are involved in this specific developmental process. However, not much is known about lncRNAs involved in human thymopoiesis. Several studies profiled either sorted thymocytes from mice or differentiation stages of mature T cells [155, 156], thereby already revealing fluctuations in lncRNA expression during T cell development.

The T-ALL oncogene *NOTCH1* is also necessary for T cell lineage commitment in the first stages of T cell development. NOTCH1 signaling is high in the CD34^+^ thymocytes but drops significantly during the β-selection process when the cells differentiate towards CD4^+^CD8^+^ double positive thymocytes. In addition to the role of NOTCH1 in lncRNA expression in T-ALL (see above), Durinck et al. also examined lncRNAs in T cell development [102]. Human thymic CD34^+^ progenitor T cells were plated on an OP9 stromal cell layer that expresses the NOTCH ligand DLL-1 (delta-like ligand 1), leading to activation of NOTCH1 signaling. RNA - sequencing was performed after 48 hours of co-culture and showed a clear shift in lncRNA expression. Furthermore, ex vivo purified human thymocyte subsets (CD34^+^CD1^−^CD4^+^, CD34^+^CD1^+^CD4^+^, CD4^+^CD8^+^CD3^−^, and CD4^+^CD8^+^CD3^+^) were profiled on an expression array that detected also lncRNAs. The NOTCH-regulated lncRNAs selected from the co-culture experiment clearly followed the expression pattern of *DTX1*, a protein-coding NOTCH1 target gene that is expressed in CD34^+^ thymocytes but not in CD4^+^CD8^+^ double positive T cells. With this study, the importance of NOTCH1 in the regulation of lncRNA expression during T cell development was clearly shown. Expression profiling of these T cell subsets also revealed several other lncRNAs with dynamic expression patterns during human T cell development, suggesting that these also have specific roles during the T cell maturation process [103].

To elucidate the molecular mechanisms that control early hematopoietic lineage choices in human, Casero et al. performed RNA - sequencing on several stages of B and T cell development [157]. Also here, stage-specific patterns of lncRNA expression were identified during the different stages of T cell development. Remarkably, cell type-specific lncRNAs, and not high expressed lncRNAs, were characterized by high densities of H3K4me1 and H3K4me3 histone modifications (marks for respectively active enhancers and promoters). Another interesting difference between lncRNAs and protein-coding genes was detected if the samples were clustered based on differentially expressed genes. For protein-coding genes, the CD34^+^ thymic progenitor cells segregated with the CD34^+^ populations in the bone marrow and not with more mature (CD34^−^) thymic progenitor cells. However, lncRNAs clearly made the distinction between thymic cells and bone marrow-derived cells. With these data, the authors could show that the cell type-specific nature of lncRNA expression could be used to define developmental relationships.

Despite the low amount of studies describing the role of miRNAs and lncRNAs in early T cell development, they already suggest that noncoding RNAs complement protein-coding genes in their ability to guide early T cell progenitors through the different maturation stages.

## Conclusions

The role for miRNAs and long noncoding RNAs has been described in several cancer entities and in developmental processes. However, it remains a challenge to define the functional activities of these noncoding RNAs, especially for long noncoding RNAs since their potential mechanism of action can be very broad. Nevertheless, the oncogenic roles for several miRNAs (ex. the *miR*-*17*~*92* cluster [23]) and lncRNAs (ex. *MALAT1*, *HOTAIR*… [52, 53]) have been described in detail for several cancer entities.

In T-ALL, the role for miRNAs is already explored in depth. One landmark publication by the Wendel team could link several miRNAs to protein-coding genes with a known tumor suppressive role in T-ALL, also showing the cooperative effect of several miRNAs on the same mRNA [121]. This paved the way for several other studies that could expand this miRNA-mRNA network. Also in T cell development, there seems to be a role for miRNAs; however, more in-depth studies should be performed to profile the miRNAs that have key roles during these developmental steps.

The role for lncRNAs in T-ALL and T cell development is less established in comparison to miRNAs. Nevertheless, the discovery of *LUNAR1*, a NOTCH1-activated lncRNA that regulates the expression of *IGF1R* in T-ALL [118], proves that there are lncRNAs involved in the oncogenic development of T-ALL. Furthermore, several studies identified lncRNAs with a specific expression pattern in T-ALL and T cell development, but the functional mechanisms of these lncRNAs have not been discovered. This is partly due to the lack of species conservation of lncRNAs, which makes it difficult to study them in mouse models, but also because a detailed study describing an in-depth and full transcriptome of all discrete stages of human T cell development is still missing. Another obstacle is the broad range of possible functional mechanisms that lncRNAs could have, which is not the case for miRNAs, and the lack of robust genetic tools in human primary hematopoietic precursors cells to functionally study the role of individual lncRNAs.

As more and more functions for miRNAs and lncRNAs are discovered, several possible pharmacological inhibitory mechanisms, for example the usage of antisense oligonucleotides, are being tested to target these noncoding RNAs [158, 159]. The interesting feature of noncoding RNAs is that their expression is more tissue specific than most protein-coding oncogenes. Drugs targeting these tissue specific RNAs could then result in less off-target effects of the therapy. Because of this, noncoding RNA research with a focus on these ectopic expressed noncoding RNAs should be further established, taken into account that there should also be a possibility to identify patients in clinic that could benefit from these specific treatments.

## Abbreviations

ASO: Antisense oligonucleotide
CD: Cluster of differentiation
ceRNA: Competitive endogenous RNA
CHART: Capture hybridization analysis of RNA targets
ChIRP: Chromatin isolation by RNA purification
CRISPR: Cluster of regularly interspaced short palindromic repeats
DLL-1: Delta-like ligand 1
eRNA: Enhancer RNA
ETP-ALL: Early T cell precursor ALL
FISH: Fluorescence in situ hybridization
GSI: γ-Secretase inhibitor
HOTAIR: Homeobox transcript antisense RNA
HPC: Hematopoietic progenitor cells
ICN1: Intracellular NOTCH1
IGF1R: Insulin-like growth factor 1 receptor
IL-3: Interleukin 3
IL-7: Interleukin 7
lncRNA: Long noncoding RNA
LUNAR1: Leukemia-induced noncoding activator RNA 1
MALAT1: Metastasis-associated lung adenocarcinoma transcript 1
miRNA: MicroRNA
NALT: NOTCH1-associated lncRNA in T-ALL
PRC2: Polycomb repressive complex 2
RAP: RNA antisense purification
RIP: RNA immunoprecipitation
RISC: RNA-induced silencing complex
T-ALL: T cell acute lymphoblastic leukemia
TCR: T cell receptor
UTR: Untranslated region
XIST: X-inactivate specific transcript

## References

[1] Djebali S, Davis CA, Merkel A, Dobin A, Lassmann T, Mortazavi A, Tanzer A, Lagarde J, Lin W, Schlesinger F (2012). Landscape of transcription in human cells. Nature.

[2] Hahn MW, Wray GA (2002). The g-value paradox. Evol Dev.

[3] Taft RJ, Pheasant M, Mattick JS (2007). The relationship between non-protein-coding DNA and eukaryotic complexity. Bioessays.

[4] Lee RC, Feinbaum RL, Ambros V (1993). The C. elegans heterochronic gene lin-4 encodes small RNAs with antisense complementarity to lin-14. Cell.

[5] Cai X, Hagedorn CH, Cullen BR (2004). Human microRNAs are processed from capped, polyadenylated transcripts that can also function as mRNAs. RNA.

[6] Lee Y, Jeon K, Lee JT, Kim S, Kim VN (2002). MicroRNA maturation: stepwise processing and subcellular localization. EMBO J.

[7] Lee Y, Ahn C, Han J, Choi H, Kim J, Yim J, Lee J, Provost P, Radmark O, Kim S, Kim VN (2003). The nuclear RNase III Drosha initiates microRNA processing. Nature.

[8] Yi R, Qin Y, Macara IG, Cullen BR (2003). Exportin-5 mediates the nuclear export of pre-microRNAs and short hairpin RNAs. Genes Dev.

[9] Lund E, Guttinger S, Calado A, Dahlberg JE, Kutay U (2004). Nuclear export of microRNA precursors. Science.

[10] Denli AM, Tops BB, Plasterk RH, Ketting RF, Hannon GJ (2004). Processing of primary microRNAs by the Microprocessor complex. Nature.

[11] Gregory RI, Yan KP, Amuthan G, Chendrimada T, Doratotaj B, Cooch N, Shiekhattar R (2004). The Microprocessor complex mediates the genesis of microRNAs. Nature.

[12] Landthaler M, Yalcin A, Tuschl T (2004). The human DiGeorge syndrome critical region gene 8 and Its D. melanogaster homolog are required for miRNA biogenesis. Curr Biol.

[13] Ha M, Kim VN (2014). Regulation of microRNA biogenesis. Nat Rev Mol Cell Biol.

[14] Vasudevan S, Tong Y, Steitz JA (2007). Switching from repression to activation: microRNAs can up-regulate translation. Science.

[15] Jansson MD, Lund AH (2012). MicroRNA and cancer. Mol Oncol.

[16] Hayes J, Peruzzi PP, Lawler S (2014). MicroRNAs in cancer: biomarkers, functions and therapy. Trends Mol Med.

[17] Lin S, Gregory RI (2015). MicroRNA biogenesis pathways in cancer. Nat Rev Cancer.

[18] Ventura A, Young AG, Winslow MM, Lintault L, Meissner A, Erkeland SJ, Newman J, Bronson RT, Crowley D, Stone JR (2008). Targeted deletion reveals essential and overlapping functions of the miR-17 through 92 family of miRNA clusters. Cell.

[19] O'Donnell KA, Wentzel EA, Zeller KI, Dang CV, Mendell JT (2005). c-Myc-regulated microRNAs modulate E2F1 expression. Nature.

[20] Schulte JH, Horn S, Otto T, Samans B, Heukamp LC, Eilers UC, Krause M, Astrahantseff K, Klein-Hitpass L, Buettner R (2008). MYCN regulates oncogenic MicroRNAs in neuroblastoma. Int J Cancer.

[21] Yan HL, Xue G, Mei Q, Wang YZ, Ding FX, Liu MF, Lu MH, Tang Y, Yu HY, Sun SH (2009). Repression of the miR-17-92 cluster by p53 has an important function in hypoxia-induced apoptosis. EMBO J.

[22] Mu P, Han YC, Betel D, Yao E, Squatrito M, Ogrodowski P, de Stanchina E, D'Andrea A, Sander C, Ventura A (2009). Genetic dissection of the miR-17~92 cluster of microRNAs in Myc-induced B-cell lymphomas. Genes Dev.

[23] Mogilyansky E, Rigoutsos I (2013). The miR-17/92 cluster: a comprehensive update on its genomics, genetics, functions and increasingly important and numerous roles in health and disease. Cell Death Differ.

[24] Calin GA, Dumitru CD, Shimizu M, Bichi R, Zupo S, Noch E, Aldler H, Rattan S, Keating M, Rai K (2002). Frequent deletions and down-regulation of micro- RNA genes miR15 and miR16 at 13q14 in chronic lymphocytic leukemia. Proc Natl Acad Sci U S A.

[25] Bonci D, Coppola V, Musumeci M, Addario A, Giuffrida R, Memeo L, D'Urso L, Pagliuca A, Biffoni M, Labbaye C (2008). The miR-15a-miR-16-1 cluster controls prostate cancer by targeting multiple oncogenic activities. Nat Med.

[26] Garzon R, Calin GA, Croce CM (2009). MicroRNAs in cancer. Annu Rev Med.

[27] Esquela-Kerscher A, Slack FJ (2006). Oncomirs—microRNAs with a role in cancer. Nat Rev Cancer.

[28] Mei Y, Clark D, Mao L (2013). Novel dimensions of piRNAs in cancer. Cancer Lett.

[29] Moyano M, Stefani G (2015). piRNA involvement in genome stability and human cancer. J Hematol Oncol.

[30] Naidu S, Magee P, Garofalo M (2015). MiRNA-based therapeutic intervention of cancer. J Hematol Oncol.

[31] Cancer Genome Atlas Research N (2013). Genomic and epigenomic landscapes of adult de novo acute myeloid leukemia. N Engl J Med.

[32] Brown CJ, Hendrich BD, Rupert JL, Lafreniere RG, Xing Y, Lawrence J, Willard HF (1992). The human XIST gene: analysis of a 17 kb inactive X-specific RNA that contains conserved repeats and is highly localized within the nucleus. Cell.

[33] Penny GD, Kay GF, Sheardown SA, Rastan S, Brockdorff N (1996). Requirement for Xist in X chromosome inactivation. Nature.

[34] Diederichs S (2014). The four dimensions of noncoding RNA conservation. Trends Genet.

[35] Johnsson P, Lipovich L, Grander D, Morris KV (2014). Evolutionary conservation of long non-coding RNAs; sequence, structure, function. Biochim Biophys Acta.

[36] Volders PJ, Verheggen K, Menschaert G, Vandepoele K, Martens L, Vandesompele J, Mestdagh P (2015). An update on LNCipedia: a database for annotated human lncRNA sequences. Nucleic Acids Res.

[37] Rinn JL, Chang HY (2012). Genome regulation by long noncoding RNAs. Annu Rev Biochem.

[38] Davidovich C, Cech TR (2015). The recruitment of chromatin modifiers by long noncoding RNAs: lessons from PRC2. RNA.

[39] Brockdorff N (2013). Noncoding RNA and Polycomb recruitment. RNA.

[40] Kino T, Hurt DE, Ichijo T, Nader N, Chrousos GP (2010). Noncoding RNA gas5 is a growth arrest- and starvation-associated repressor of the glucocorticoid receptor. Sci Signal.

[41] Martianov I, Ramadass A, Serra Barros A, Chow N, Akoulitchev A (2007). Repression of the human dihydrofolate reductase gene by a non-coding interfering transcript. Nature.

[42] Mariner PD, Walters RD, Espinoza CA, Drullinger LF, Wagner SD, Kugel JF, Goodrich JA (2008). Human Alu RNA is a modular transacting repressor of mRNA transcription during heat shock. Mol Cell.

[43] Li W, Notani D, Rosenfeld MG (2016). Enhancers as non-coding RNA transcription units: recent insights and future perspectives. Nat Rev Genet.

[44] Lam MT, Li W, Rosenfeld MG, Glass CK (2014). Enhancer RNAs and regulated transcriptional programs. Trends Biochem Sci.

[45] Beltran M, Puig I, Pena C, Garcia JM, Alvarez AB, Pena R, Bonilla F, de Herreros AG (2008). A natural antisense transcript regulates Zeb2/Sip1 gene expression during Snail1-induced epithelial-mesenchymal transition. Genes Dev.

[46] Munroe SH, Lazar MA (1991). Inhibition of c-erbA mRNA splicing by a naturally occurring antisense RNA. J Biol Chem.

[47] Poliseno L, Salmena L, Zhang J, Carver B, Haveman WJ, Pandolfi PP (2010). A coding-independent function of gene and pseudogene mRNAs regulates tumour biology. Nature.

[48] Yu G, Yao W, Gumireddy K, Li A, Wang J, Xiao W, Chen K, Xiao H, Li H, Tang K (2014). Pseudogene PTENP1 functions as a competing endogenous RNA to suppress clear-cell renal cell carcinoma progression. Mol Cancer Ther.

[49] Sanchez-Mejias A, Tay Y (2015). Competing endogenous RNA networks: tying the essential knots for cancer biology and therapeutics. J Hematol Oncol.

[50] Geisler S, Coller J (2013). RNA in unexpected places: long non-coding RNA functions in diverse cellular contexts. Nat Rev Mol Cell Biol.

[51] Maass PG, Luft FC, Bahring S (2014). Long non-coding RNA in health and disease. J Mol Med (Berl).

[52] Gutschner T, Hammerle M, Diederichs S (2013). MALAT1—a paradigm for long noncoding RNA function in cancer. J Mol Med (Berl).

[53] Gupta RA, Shah N, Wang KC, Kim J, Horlings HM, Wong DJ, Tsai MC, Hung T, Argani P, Rinn JL (2010). Long non-coding RNA HOTAIR reprograms chromatin state to promote cancer metastasis. Nature.

[54] Kim TK, Hemberg M, Gray JM, Costa AM, Bear DM, Wu J, Harmin DA, Laptewicz M, Barbara-Haley K, Kuersten S (2010). Widespread transcription at neuronal activity-regulated enhancers. Nature.

[55] Lai F, Shiekhattar R (2014). Enhancer RNAs: the new molecules of transcription. Curr Opin Genet Dev.

[56] Paralkar VR, Taborda CC, Huang P, Yao Y, Kossenkov AV, Prasad R, Luan J, Davies JO, Hughes JR, Hardison RC (2016). Unlinking an lncRNA from its associated cis element. Mol Cell.

[57] Engreitz JM, Haines JE, Perez EM, Munson G, Chen J, Kane M, McDonel PE, Guttman M, Lander ES (2016). Local regulation of gene expression by lncRNA promoters, transcription and splicing. Nature.

[58] Taghon T, Waegemans E, Van de Walle I (2012). Notch signaling during human T cell development. Curr Top Microbiol Immunol.

[59] Dik WA, Pike-Overzet K, Weerkamp F, de Ridder D, de Haas EF, Baert MR, van der Spek P, Koster EE, Reinders MJ, van Dongen JJ (2005). New insights on human T cell development by quantitative T cell receptor gene rearrangement studies and gene expression profiling. J Exp Med.

[60] Germain RN (2002). T-cell development and the CD4-CD8 lineage decision. Nat Rev Immunol.

[61] Van de Walle I, De Smet G, De Smedt M, Vandekerckhove B, Leclercq G, Plum J, Taghon T (2009). An early decrease in Notch activation is required for human TCR-alphabeta lineage differentiation at the expense of TCR-gammadelta T cells. Blood.

[62] Van de Walle I, Dolens AC, Durinck K, De Mulder K, Van Loocke W, Damle S, Waegemans E, De Medts J, Velghe I, De Smedt M (2016). GATA3 induces human T-cell commitment by restraining Notch activity and repressing NK-cell fate. Nat Commun.

[63] Blom B, Spits H (2006). Development of human lymphoid cells. Annu Rev Immunol.

[64] Garcia-Peydro M, de Yebenes VG, Toribio ML (2003). Sustained Notch1 signaling instructs the earliest human intrathymic precursors to adopt a gammadelta T-cell fate in fetal thymus organ culture. Blood.

[65] Jones ME, Zhuang Y (2007). Acquisition of a functional T cell receptor during T lymphocyte development is enforced by HEB and E2A transcription factors. Immunity.

[66] Yui MA, Rothenberg EV (2014). Developmental gene networks: a triathlon on the course to T cell identity. Nat Rev Immunol.

[67] Rothenberg EV, Kueh HY, Yui MA, Zhang JA (2016). Hematopoiesis and T-cell specification as a model developmental system. Immunol Rev.

[68] Rothenberg EV, Ungerback J, Champhekar A (2016). Forging T-lymphocyte identity: intersecting networks of transcriptional control. Adv Immunol.

[69] Koch U, Radtke F (2011). Mechanisms of T cell development and transformation. Annu Rev Cell Dev Biol.

[70] Ferrando AA, Neuberg DS, Staunton J, Loh ML, Huard C, Raimondi SC, Behm FG, Pui CH, Downing JR, Gilliland DG (2002). Gene expression signatures define novel oncogenic pathways in T cell acute lymphoblastic leukemia. Cancer Cell.

[71] Meijerink JP (2010). Genetic rearrangements in relation to immunophenotype and outcome in T-cell acute lymphoblastic leukaemia. Best Pract Res Clin Haematol.

[72] Van Vlierberghe P, Pieters R, Beverloo HB, Meijerink JP (2008). Molecular-genetic insights in paediatric T-cell acute lymphoblastic leukaemia. Br J Haematol.

[73] Soulier J, Clappier E, Cayuela JM, Regnault A, Garcia-Peydro M, Dombret H, Baruchel A, Toribio ML, Sigaux F (2005). HOXA genes are included in genetic and biologic networks defining human acute T-cell leukemia (T-ALL). Blood.

[74] Homminga I, Pieters R, Langerak AW, de Rooi JJ, Stubbs A, Verstegen M, Vuerhard M, Buijs-Gladdines J, Kooi C, Klous P (2011). Integrated transcript and genome analyses reveal NKX2-1 and MEF2C as potential oncogenes in T cell acute lymphoblastic leukemia. Cancer Cell.

[75] Weng AP, Ferrando AA, Lee W, Morris JP, Silverman LB, Sanchez-Irizarry C, Blacklow SC, Look AT, Aster JC (2004). Activating mutations of NOTCH1 in human T cell acute lymphoblastic leukemia. Science.

[76] Tzoneva G, Ferrando AA (2012). Recent advances on NOTCH signaling in T-ALL. Curr Top Microbiol Immunol.

[77] Karrman K, Johansson B. Pediatric T-cell acute lymphoblastic leukemia. Genes Chromosomes Cancer. 2017;56:89-116. 10.1002/gcc.22416 27636224

[78] Van Vlierberghe P, Ferrando A (2012). The molecular basis of T cell acute lymphoblastic leukemia. J Clin Invest.

[79] Wei S, Wang K (2015). Long noncoding RNAs: pivotal regulators in acute myeloid leukemia. Exp Hematol Oncol.

[80] Morlando M, Ballarino M, Fatica A (2015). Long non-coding RNAs: new players in hematopoiesis and leukemia. Front Med (Lausanne).

[81] Zhang X, Hu W: Long noncoding RNAs in hematopoiesis. F1000Res 2016, 5. 10.12688/f1000research.8349.1 PMC4955016 27508063

[82] Satpathy AT, Chang HY (2015). Long noncoding RNA in hematopoiesis and immunity. Immunity.

[83] Schotte D, Pieters R, Den Boer ML (2012). MicroRNAs in acute leukemia: from biological players to clinical contributors. Leukemia.

[84] Zhao H, Wang D, Du W, Gu D, Yang R (2010). MicroRNA and leukemia: tiny molecule, great function. Crit Rev Oncol Hematol.

[85] Montagner S, Deho L, Monticelli S (2014). MicroRNAs in hematopoietic development. BMC Immunol.

[86] Zhang H, Yang JH, Zheng YS, Zhang P, Chen X, Wu J, Xu L, Luo XQ, Ke ZY, Zhou H (2009). Genome-wide analysis of small RNA and novel MicroRNA discovery in human acute lymphoblastic leukemia based on extensive sequencing approach. PLoS One.

[87] Schotte D, Akbari Moqadam F, Lange-Turenhout EA, Chen C, van Ijcken WF, Pieters R, den Boer ML (2011). Discovery of new microRNAs by small RNAome deep sequencing in childhood acute lymphoblastic leukemia. Leukemia.

[88] Mestdagh P, Hartmann N, Baeriswyl L, Andreasen D, Bernard N, Chen C, Cheo D, D'Andrade P, DeMayo M, Dennis L (2014). Evaluation of quantitative miRNA expression platforms in the microRNA quality control (miRQC) study. Nat Methods.

[89] Wong N, Wang X (2015). miRDB: an online resource for microRNA target prediction and functional annotations. Nucleic Acids Res.

[90] Betel D, Wilson M, Gabow A, Marks DS, Sander C (2008). The microRNA.org resource: targets and expression. Nucleic Acids Res.

[91] Agarwal V, Bell GW, Nam JW, Bartel DP: Predicting effective microRNA target sites in mammalian mRNAs. Elife 2015, 4 10.7554/eLife.05005 PMC4532895 26267216

[92] Van Peer G, De Paepe A, Stock M, Anckaert J, Volders PJ, Vandesompele J, De Baets B, Waegeman W. miSTAR: miRNA target prediction through modeling quantitative and qualitative miRNA binding site information in a stacked model structure. Nucleic Acids Res. 2016; Epub ahead of print. 10.1093/nar/gkw1260 PMC5397177 27986855

[93] Helwak A, Kudla G, Dudnakova T, Tollervey D (2013). Mapping the human miRNA interactome by CLASH reveals frequent noncanonical binding. Cell.

[94] Thomson DW, Bracken CP, Goodall GJ (2011). Experimental strategies for microRNA target identification. Nucleic Acids Res.

[95] Licatalosi DD, Mele A, Fak JJ, Ule J, Kayikci M, Chi SW, Clark TA, Schweitzer AC, Blume JE, Wang X (2008). HITS-CLIP yields genome-wide insights into brain alternative RNA processing. Nature.

[96] Hafner M, Landthaler M, Burger L, Khorshid M, Hausser J, Berninger P, Rothballer A, Ascano M, Jungkamp AC, Munschauer M (2010). Transcriptome-wide identification of RNA-binding protein and microRNA target sites by PAR-CLIP. Cell.

[97] Chi SW, Zang JB, Mele A, Darnell RB (2009). Argonaute HITS-CLIP decodes microRNA-mRNA interaction maps. Nature.

[98] Tan LP, Seinen E, Duns G, de Jong D, Sibon OC, Poppema S, Kroesen BJ, Kok K, van den Berg A (2009). A high throughput experimental approach to identify miRNA targets in human cells. Nucleic Acids Res.

[99] Mets E, Van Peer G, Van der Meulen J, Boice M, Taghon T, Goossens S, Mestdagh P, Benoit Y, De Moerloose B, Van Roy N (2014). MicroRNA-128-3p is a novel oncomiR targeting PHF6 in T-cell acute lymphoblastic leukemia. Haematologica.

[100] Pear WS, Aster JC, Scott ML, Hasserjian RP, Soffer B, Sklar J, Baltimore D (1996). Exclusive development of T cell neoplasms in mice transplanted with bone marrow expressing activated Notch alleles. J Exp Med.

[101] Junker F, Chabloz A, Koch U, Radtke F (2015). Dicer1 imparts essential survival cues in Notch-driven T-ALL via miR-21-mediated tumor suppressor Pdcd4 repression. Blood.

[102] Durinck K, Wallaert A, Van de Walle I, Van Loocke W, Volders PJ, Vanhauwaert S, Geerdens E, Benoit Y, Van Roy N, Poppe B (2014). The Notch driven long non-coding RNA repertoire in T-cell acute lymphoblastic leukemia. Haematologica.

[103] Wallaert A, Durinck K, Van Loocke W, Van de Walle I, Matthijssens F, Volders PJ, Avila Cobos F, Rombaut D, Rondou P, Mestdagh P, et al. Long noncoding RNA signatures define oncogenic subtypes in T-cell acute lymphoblastic leukemia. Leukemia. 2016;30:1927-30. 10.1038/leu.2016.82 27168467

[104] Derrien T, Johnson R, Bussotti G, Tanzer A, Djebali S, Tilgner H, Guernec G, Martin D, Merkel A, Knowles DG (2012). The GENCODE v7 catalog of human long noncoding RNAs: analysis of their gene structure, evolution, and expression. Genome Res.

[105] Cui P, Lin Q, Ding F, Xin C, Gong W, Zhang L, Geng J, Zhang B, Yu X, Yang J (2010). A comparison between ribo-minus RNA-sequencing and polyA-selected RNA-sequencing. Genomics.

[106] Mortazavi A, Williams BA, McCue K, Schaeffer L, Wold B (2008). Mapping and quantifying mammalian transcriptomes by RNA-Seq. Nat Methods.

[107] Guttman M, Amit I, Garber M, French C, Lin MF, Feldser D, Huarte M, Zuk O, Carey BW, Cassady JP (2009). Chromatin signature reveals over a thousand highly conserved large non-coding RNAs in mammals. Nature.

[108] Hung T, Wang Y, Lin MF, Koegel AK, Kotake Y, Grant GD, Horlings HM, Shah N, Umbricht C, Wang P (2011). Extensive and coordinated transcription of noncoding RNAs within cell-cycle promoters. Nat Genet.

[109] Chu C, Qu K, Zhong FL, Artandi SE, Chang HY (2011). Genomic maps of long noncoding RNA occupancy reveal principles of RNA-chromatin interactions. Mol Cell.

[110] Simon MD, Wang CI, Kharchenko PV, West JA, Chapman BA, Alekseyenko AA, Borowsky ML, Kuroda MI, Kingston RE (2011). The genomic binding sites of a noncoding RNA. Proc Natl Acad Sci U S A.

[111] Engreitz J, Lander ES, Guttman M (2015). RNA antisense purification (RAP) for mapping RNA interactions with chromatin. Methods Mol Biol.

[112] Gilbert C, Svejstrup JQ: RNA immunoprecipitation for determining RNA-protein associations in vivo. Curr Protoc Mol Biol 2006, Chapter 27:Unit 27 24. 10.1002/0471142727.mb2704s75 18265380

[113] Chu C, Spitale RC, Chang HY (2015). Technologies to probe functions and mechanisms of long noncoding RNAs. Nat Struct Mol Biol.

[114] Qi LS, Larson MH, Gilbert LA, Doudna JA, Weissman JS, Arkin AP, Lim WA (2013). Repurposing CRISPR as an RNA-guided platform for sequence-specific control of gene expression. Cell.

[115] Pang KC, Frith MC, Mattick JS (2006). Rapid evolution of noncoding RNAs: lack of conservation does not mean lack of function. Trends Genet.

[116] Amaral PP, Leonardi T, Han N, Vire E, Gascoigne DK, Arias-Carrasco R, Buscher M, Zhang A, Pluchino S, Maracaja-Coutinho V, et al: Genomic positional conservation identifies topological anchor point (tap)RNAs linked to developmental loci. bioRxiv 2016;051052. 10.1186/s13059-018-1405-5 PMC5853149 29540241

[117] Ulitsky I (2016). Evolution to the rescue: using comparative genomics to understand long non-coding RNAs. Nat Rev Genet.

[118] Trimarchi T, Bilal E, Ntziachristos P, Fabbri G, Dalla-Favera R, Tsirigos A, Aifantis I (2014). Genome-wide mapping and characterization of Notch-regulated long noncoding RNAs in acute leukemia. Cell.

[119] Mavrakis KJ, Wolfe AL, Oricchio E, Palomero T, de Keersmaecker K, McJunkin K, Zuber J, James T, Khan AA, Leslie CS (2010). Genome-wide RNA-mediated interference screen identifies miR-19 targets in Notch-induced T-cell acute lymphoblastic leukaemia. Nat Cell Biol.

[120] Ye H, Liu X, Lv M, Wu Y, Kuang S, Gong J, Yuan P, Zhong Z, Li Q, Jia H (2012). MicroRNA and transcription factor co-regulatory network analysis reveals miR-19 inhibits CYLD in T-cell acute lymphoblastic leukemia. Nucleic Acids Res.

[121] Mavrakis KJ, Van Der Meulen J, Wolfe AL, Liu X, Mets E, Taghon T, Khan AA, Setty M, Rondou P, Vandenberghe P (2011). A cooperative microRNA-tumor suppressor gene network in acute T-cell lymphoblastic leukemia (T-ALL). Nat Genet.

[122] Mansour MR, Sanda T, Lawton LN, Li X, Kreslavsky T, Novina CD, Brand M, Gutierrez A, Kelliher MA, Jamieson CH (2013). The TAL1 complex targets the FBXW7 tumor suppressor by activating miR-223 in human T cell acute lymphoblastic leukemia. J Exp Med.

[123] Correia NC, Durinck K, Leite AP, Ongenaert M, Rondou P, Speleman F, Enguita FJ, Barata JT (2013). Novel TAL1 targets beyond protein-coding genes: identification of TAL1-regulated microRNAs in T-cell acute lymphoblastic leukemia. Leukemia.

[124] Gusscott S, Kuchenbauer F, Humphries RK, Weng AP (2012). Notch-mediated repression of miR-223 contributes to IGF1R regulation in T-ALL. Leuk Res.

[125] Lv M, Zhang X, Jia H, Li D, Zhang B, Zhang H, Hong M, Jiang T, Jiang Q, Lu J (2012). An oncogenic role of miR-142-3p in human T-cell acute lymphoblastic leukemia (T-ALL) by targeting glucocorticoid receptor-alpha and cAMP/PKA pathways. Leukemia.

[126] Fan SJ, Li HB, Cui G, Kong XL, Sun LL, Zhao YQ, Li YH, Zhou J (2016). miRNA-149* promotes cell proliferation and suppresses apoptosis by mediating JunB in T-cell acute lymphoblastic leukemia. Leuk Res.

[127] Miao MH, Ji XQ, Zhang H, Xu J, Zhu H, Shao XJ: miR-590 promotes cell proliferation and invasion in T-cell acute lymphoblastic leukaemia by inhibiting RB1. Oncotarget. 2016;7:39527-39534. 10.18632/oncotarget.8414 PMC5129950 27036041

[128] Verduci L, Azzalin G, Gioiosa S, Carissimi C, Laudadio I, Fulci V, Macino G (2015). microRNA-181a enhances cell proliferation in acute lymphoblastic leukemia by targeting EGR1. Leuk Res.

[129] Fragoso R, Mao T, Wang S, Schaffert S, Gong X, Yue S, Luong R, Min H, Yashiro-Ohtani Y, Davis M (2012). Modulating the strength and threshold of NOTCH oncogenic signals by mir-181a-1/b-1. PLoS Genet.

[130] Sanghvi VR, Mavrakis KJ, Van der Meulen J, Boice M, Wolfe AL, Carty M, Mohan P, Rondou P, Socci ND, Benoit Y (2014). Characterization of a set of tumor suppressor microRNAs in T cell acute lymphoblastic leukemia. Sci Signal.

[131] Mets E, Van der Meulen J, Van Peer G, Boice M, Mestdagh P, Van de Walle I, Lammens T, Goossens S, De Moerloose B, Benoit Y (2015). MicroRNA-193b-3p acts as a tumor suppressor by targeting the MYB oncogene in T-cell acute lymphoblastic leukemia. Leukemia.

[132] Yin JJ, Liang B, Zhan XR (2015). MicroRNA-204 inhibits cell proliferation in T-cell acute lymphoblastic leukemia by down-regulating SOX4. Int J Clin Exp Pathol.

[133] Nagel S, Venturini L, Przybylski GK, Grabarczyk P, Schmidt CA, Meyer C, Drexler HG, Macleod RA, Scherr M (2009). Activation of miR-17-92 by NK-like homeodomain proteins suppresses apoptosis via reduction of E2F1 in T-cell acute lymphoblastic leukemia. Leuk Lymphoma.

[134] Schotte D, Lange-Turenhout EA, Stumpel DJ, Stam RW, Buijs-Gladdines JG, Meijerink JP, Pieters R, Den Boer ML (2010). Expression of miR-196b is not exclusively MLL-driven but is especially linked to activation of HOXA genes in pediatric acute lymphoblastic leukemia. Haematologica.

[135] Coskun E, von der Heide EK, Schlee C, Kuhnl A, Gokbuget N, Hoelzer D, Hofmann WK, Thiel E, Baldus CD (2011). The role of microRNA-196a and microRNA-196b as ERG regulators in acute myeloid leukemia and acute T-lymphoblastic leukemia. Leuk Res.

[136] Chiaretti S, Messina M, Tavolaro S, Zardo G, Elia L, Vitale A, Fatica A, Gorello P, Piciocchi A, Scappucci G (2010). Gene expression profiling identifies a subset of adult T-cell acute lymphoblastic leukemia with myeloid-like gene features and over-expression of miR-223. Haematologica.

[137] Coskun E, Neumann M, Schlee C, Liebertz F, Heesch S, Goekbuget N, Hoelzer D, Baldus CD (2013). MicroRNA profiling reveals aberrant microRNA expression in adult ETP-ALL and functional studies implicate a role for miR-222 in acute leukemia. Leuk Res.

[138] Li X, Sanda T, Look AT, Novina CD, von Boehmer H (2011). Repression of tumor suppressor miR-451 is essential for NOTCH1-induced oncogenesis in T-ALL. J Exp Med.

[139] Ortega M, Bhatnagar H, Lin AP, Wang L, Aster JC, Sill H, Aguiar RC (2015). A microRNA-mediated regulatory loop modulates NOTCH and MYC oncogenic signals in B- and T-cell malignancies. Leukemia.

[140] Chang TC, Yu D, Lee YS, Wentzel EA, Arking DE, West KM, Dang CV, Thomas-Tikhonenko A, Mendell JT (2008). Widespread microRNA repression by Myc contributes to tumorigenesis. Nat Genet.

[141] Kumar V, Palermo R, Talora C, Campese AF, Checquolo S, Bellavia D, Tottone L, Testa G, Miele E, Indraccolo S (2014). Notch and NF-kB signaling pathways regulate miR-223/FBXW7 axis in T-cell acute lymphoblastic leukemia. Leukemia.

[142] Correia NC, Melao A, Povoa V, Sarmento L, Gomez de Cedron M, Malumbres M, Enguita FJ, Barata JT (2016). microRNAs regulate TAL1 expression in T-cell acute lymphoblastic leukemia. Oncotarget.

[143] Wang Y, Wu P, Lin R, Rong L, Xue Y, Fang Y (2015). LncRNA NALT interaction with NOTCH1 promoted cell proliferation in pediatric T cell acute lymphoblastic leukemia. Sci Rep.

[144] Volders PJ, Helsens K, Wang X, Menten B, Martens L, Gevaert K, Vandesompele J, Mestdagh P (2013). LNCipedia: a database for annotated human lncRNA transcript sequences and structures. Nucleic Acids Res.

[145] Cobb BS, Nesterova TB, Thompson E, Hertweck A, O'Connor E, Godwin J, Wilson CB, Brockdorff N, Fisher AG, Smale ST, Merkenschlager M (2005). T cell lineage choice and differentiation in the absence of the RNase III enzyme Dicer. J Exp Med.

[146] Chong MM, Zhang G, Cheloufi S, Neubert TA, Hannon GJ, Littman DR (2010). Canonical and alternate functions of the microRNA biogenesis machinery. Genes Dev.

[147] Muljo SA, Ansel KM, Kanellopoulou C, Livingston DM, Rao A, Rajewsky K (2005). Aberrant T cell differentiation in the absence of Dicer. J Exp Med.

[148] Kirigin FF, Lindstedt K, Sellars M, Ciofani M, Low SL, Jones L, Bell F, Pauli F, Bonneau R, Myers RM (2012). Dynamic microRNA gene transcription and processing during T cell development. J Immunol.

[149] Neilson JR, Zheng GX, Burge CB, Sharp PA (2007). Dynamic regulation of miRNA expression in ordered stages of cellular development. Genes Dev.

[150] Ebert PJ, Jiang S, Xie J, Li QJ, Davis MM (2009). An endogenous positively selecting peptide enhances mature T cell responses and becomes an autoantigen in the absence of microRNA miR-181a. Nat Immunol.

[151] Li QJ, Chau J, Ebert PJ, Sylvester G, Min H, Liu G, Braich R, Manoharan M, Soutschek J, Skare P (2007). miR-181a is an intrinsic modulator of T cell sensitivity and selection. Cell.

[152] Xiao C, Srinivasan L, Calado DP, Patterson HC, Zhang B, Wang J, Henderson JM, Kutok JL, Rajewsky K (2008). Lymphoproliferative disease and autoimmunity in mice with increased miR-17-92 expression in lymphocytes. Nat Immunol.

[153] Regelin M, Blume J, Pommerencke J, Vakilzadeh R, Witzlau K, Lyszkiewicz M, Zietara N, Saran N, Schambach A, Krueger A (2015). Responsiveness of developing T cells to IL-7 signals is sustained by miR-17 approximately 92. J Immunol.

[154] Blevins R, Bruno L, Carroll T, Elliott J, Marcais A, Loh C, Hertweck A, Krek A, Rajewsky N, Chen CZ (2015). microRNAs regulate cell-to-cell variability of endogenous target gene expression in developing mouse thymocytes. PLoS Genet.

[155] Hu G, Tang Q, Sharma S, Yu F, Escobar TM, Muljo SA, Zhu J, Zhao K (2013). Expression and regulation of intergenic long noncoding RNAs during T cell development and differentiation. Nat Immunol.

[156] Ranzani V, Rossetti G, Panzeri I, Arrigoni A, Bonnal RJ, Curti S, Gruarin P, Provasi E, Sugliano E, Marconi M (2015). The long intergenic noncoding RNA landscape of human lymphocytes highlights the regulation of T cell differentiation by linc-MAF-4. Nat Immunol.

[157] Casero D, Sandoval S, Seet CS, Scholes J, Zhu Y, Ha VL, Luong A, Parekh C, Crooks GM (2015). Long non-coding RNA profiling of human lymphoid progenitor cells reveals transcriptional divergence of B cell and T cell lineages. Nat Immunol.

[158] Parasramka MA, Maji S, Matsuda A, Yan IK, Patel T (2016). Long non-coding RNAs as novel targets for therapy in hepatocellular carcinoma. Pharmacol Ther.

[159] Christopher AF, Kaur RP, Kaur G, Kaur A, Gupta V, Bansal P (2016). MicroRNA therapeutics: discovering novel targets and developing specific therapy. Perspect Clin Res.

[160] Van Peer G, Lefever S, Anckaert J, Beckers A, Rihani A, Van Goethem A, Volders PJ, Zeka F, Ongenaert M, Mestdagh P, Vandesompele J: miRBase Tracker: keeping track of microRNA annotation changes. Database (Oxford) 2014;2014. 10.1093/database/bau080 PMC4142392 25157074

